# Single points of access to fill the “GAP” for unattached patients in primary care: What makes paediatric users more likely to require a medical appointment?

**DOI:** 10.1177/08404704231215698

**Published:** 2023-12-08

**Authors:** Sebastien J. Bergeron, Catherine Lamoureux-Lamarche, David-Martin Milot, Mathieu Lanthier-Veilleux, Djamal Berbiche, Mylaine Breton

**Affiliations:** 1 198734 Université de Sherbrooke, Longueuil, Quebec, Canada.; 2 8202 Maisonneuve-Rosemont Hospital, Montreal, Quebec, Canada.

## Abstract

Unattachment to a regular primary care professional can affect children’s and adolescents’ well-being, considering their unique health needs. Having no alternative, many turn to emergency departments for non-urgent conditions. To help unattached patients access healthcare services while on waitlists, Quebec’s government implemented single access points in each administrative region across the province. Our study aimed to describe the paediatric population using single access points and identify associations between their characteristics and need for a medical appointment. Clinical-administrative data of 1,323 paediatric access point users in the Montérégie region from November 2022 to March 2023 were utilized to conduct bivariate and multivariable regression analyses. Our study showed that young age, assessment trajectory, and specific reasons for calling were more likely to necessitate a medical appointment. While access points improve accessibility to doctors, questions remain regarding the relevance of medical consultations, inequities, and possible security issues resulting from the overall process.

## Introduction

Attachment to a regular Primary Care Professional (PCP), whether a family doctor or Nurse Practitioner (NP), is an important element of Primary Healthcare (PHC), as it represents patients’ main gateway to the healthcare system.^1-3^ In contrast, unattachment leads to delays in accessing care, poorer chronic disease management, unnecessary hospitalizations and visits to the emergency department, and health inequities, as well as higher healthcare costs, morbidity, and mortality rates.^1-3^

Twenty-two percent of Canadians do not have a regular PCP.^4^ To help unattached patients find one, the 10 Canadian provinces progressively implemented Centralized Waitlists (CWLs).^5^ These lists aim to centralize these patients’ requests to access a PCP in each area and match them based on availability of the primary care workforce and, to some extent, medical needs.^6^ Although this is a first step towards better healthcare accessibility, delays for attachment to a PCP vary considerably among and within jurisdictions, with “healthy” patients waiting up to six years before being attached to a family doctor or NP in Quebec.^7^

To help CWL-registered patients access health services while awaiting attachment to a PCP, Quebec’s Ministry of Health and Social Services mandated all 18 regions across the province to implement “single points of access” (referred to as *Guichets d’accès à la première ligne* or GAPs) in 2022.^8,9^ The goal was to direct patients to the most appropriate professional or service according to their needs, within the context of a shortage of family physicians.^10^ To our knowledge, single points of access for unattached patients exist only in Quebec, and despite significant investments to implement them, no study has yet been carried out to assess them. Moreover, around 30% of Quebecers remain without a regular PCP.^4,11^

Single points of access could help improve access to PHC for paediatric patients in Quebec, considering that 35% of infants, 25% of preschoolers, and 26% of older children and adolescents remain unattached.^11,12^ Paediatric PHC is essential, as children and adolescents have specific needs with regards to growth, development, learning, immunization, and age-appropriate counselling and screening.^13,14^ Furthermore, paediatric patients represent 22% of emergency department visits in Quebec,^15^ a proportion bound to rise during seasons of high virus circulation in the context of inaccessible primary care.^16-18^

Given the significant population of children and adolescents without a regular PCP and the limited data on the single points of access recently implemented across the province, our study will fill gaps in the literature (1) by providing data on the sociodemographic and clinical characteristics of the paediatric population using the GAP service and (2) by identifying the characteristics that make some paediatric patients more prone to require a medical appointment at the end of the assessment process. Such information is relevant to decision- and policy-makers who intend to implement a similar initiative in their jurisdiction.

## Methods

### Context of the study

Located on the south shore of Montreal, Montérégie represents the second most populous region of Quebec with approximately 1,476,000 inhabitants, 20% of whom are under age 18.^19^ As of March 2023, 28% of the population was unattached to a regular PCP,^11,20^ of which 53% were registered on the CWL. Montérégie is composed of three *Centres intégrés de santé et de services sociaux* (CISSS), each administering healthcare services in their respective territory.^21^

**Figure 1.**
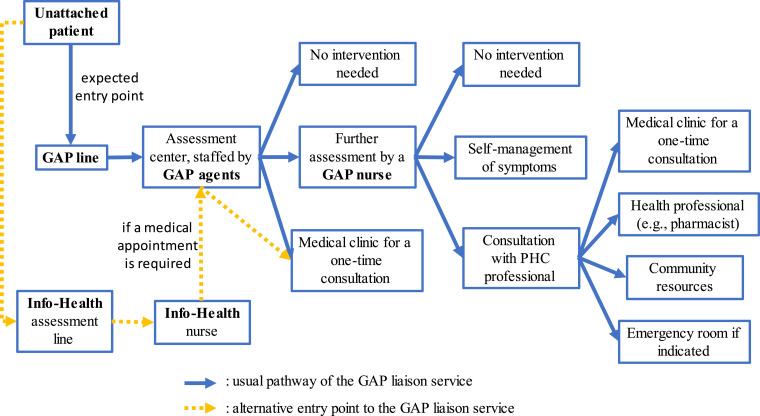
Assessment trajectories shared by the two GAPs under study. **Abbreviations**: GAP, *Guichet d’accès à la première ligne* (single point of access); PHC, primary healthcare.

The current study was conducted in one CISSS, covering two single points of access (GAPs), which can be used strictly by unattached patients in their territory who are registered on the regional waitlist. Users call the GAP liaison service, which is operated by administrative clerks (agents) and clinical nurses whose mandates are to identify patients’ needs, evaluate the urgency of the situation, provide information, and direct them towards the most appropriate PHC services when needed.^10^ General information on the functioning and assessment trajectories shared by the two GAPs in 2023 (Figure 1) were validated with GAP experts. Only GAP agents and nurses can assign a medical appointment to patients within 24 to 36 hours. Agents determine whether a medical evaluation is needed using a decision-making algorithm, while GAP nurses rely on a similar tool and their clinical judgement. Otherwise, no standardized training regarding paediatric conditions is offered to the staff. Finally, unattached patients assessed by nurses from the provincial Info-Health line and requiring a medical appointment are redirected to a GAP agent, who will offer them one if available.

### Sample

We conducted a cross-sectional study using the clinical-administrative databases of two GAPs in Montérégie, Quebec. The sample included individuals aged 0 to 17 years residing in the same health region who used the GAP liaison service between November 20, 2022 and March 31, 2023.

### Measures

Our main outcome was whether a medical appointment was deemed necessary (yes/no) at least once by the GAP service during the study period. For feasibility purposes and because only 15% of users called more than once during the study period, we proceeded as follows. For patients who did not require a medical consultation during this period, only data from the first call were retained. For those who needed a medical appointment at least once, we only kept data from the first call that led to that outcome.

The independent variables studied are detailed elsewhere. They included individual sociodemographic (sex, age, and social and material deprivation indexes based on postal code) and clinical factors (health vulnerability code, reason for call, and system affected in the event of an acute health problem), as well as characteristics related to the GAP itself (GAP territory and gateway to access).

### Analysis

Using SPSS (version 28.0.1.0) software, we performed descriptive and bivariate analyses (chi-squared and Student’s t-tests) to describe the overall study population and whether a medical consultation was deemed necessary. To assess the association between study variables and our main outcome, we built a multivariable logistic regression model (stepwise approach) including only the significant variables (*P* ≤ .05) identified in bivariate analyses.

## Results

### Objective 1: Describe the sociodemographic and clinical characteristics of the paediatric population using GAPs

Table 1 describes our study population. Overall, 1,323 paediatric patients used the GAP liaison service during the study period, with a mean of 1.2 call per user. Mean age was 7.5 years, while the youngest patient was 9 days old. Approximately one-third of patients were infants (31.7%). The majority (60.2%) had a low priority code for family doctor attribution (D or E) and no assigned health code vulnerability (96.8%).

**Table 1.** Characteristics of the overall sample and according to the requirement for a medical referral (*n* = 1,323).

	Missing data n (%)	Overall sample n (%)	Medical referral		*P* value
			Not required (n = 244)	Required (n = 1,079)	
**Sociodemographic factors**					
Sex	7 (1)				
Female		663 (50)	126 (19)	537 (81)	.611
Male		653 (50)	117 (18)	536 (82)	
Age group	0 (0)				
Infancy (0-2)		419 (32)	61 (15)	359 (85)	.010
Early childhood (3-5)		185 (14)	28 (15)	158 (85)	
Late childhood (6-11)		361 (27)	73 (20)	287 (80)	
Adolescence (12-17)		358 (27)	82 (23)	275 (77)	
Social deprivation index	13 (1)				
1 (most privileged)		341 (26)	71 (21)	270 (79)	.251
2		229 (18)	48 (21)	181 (79)	
3		261 (20)	46 (18)	215 (82)	
4		232 (18)	42 (18)	190 (82)	
5 (least privileged)		247 (19)	35 (14)	212 (86)	
Material deprivation index	13 (1)				
1 (most privileged)		291 (22)	51 (18)	240 (82)	.769
2		327 (25)	66 (20)	261 (80)	
3		285 (22)	48 (17)	237 (83)	
4		220 (17)	39 (17)	181 (83)	
5 (least privileged)		187 (14)	38 (20)	149 (80)	
**Clinical information**					
Priority code for family doctor attribution	227 (17)				
A (highest priority)		18 (2)	3 (17)	15 (83)	.271
B		5 (1)	2 (40)	3 (60)	
C		413 (38)	64 (15)	349 (85)	
D		127 (12)	21 (16)	106 (84)	
E (lowest priority)		533 (49)	107 (20)	426 (80)	
Health vulnerability code	14 (1)				
No		1,267 (97)	240 (19)	1,027 (81)	.050
Yes		42 (3)	3 (7)	39 (93)	
Main reason for calling	0 (0)				
Acute health problem		1,104 (83)	133 (12)	971 (88)	<.001
Paediatric follow-up		88 (7)	24 (27)	64 (73)	
Medication renewal or reassessment		37 (3)	2 (5)	35 (95)	
Other		94 (8)	85 (90)	9 (10)	
System affected in the event of an acute health problem	303 (27)	n = 801^a^			
Ear, nose and throat		271 (34)	34 (12)	237 (88)	.364
Respiratory		107 (13)	19 (18)	88 (82)	.244
Gastrointestinal		83 (10)	13 (16)	70 (84)	.667
Dermatological		82 (10)	7 (9)	75 (91)	.126
Infectious		59 (7)	9 (15)	50 (85)	.793
Neurodevelopmental and cognitive		55 (7)	5 (9)	50 (91)	.268
Mental health		46 (6)	4 (9)	42 (91)	.277
Other		150 (19)	29 (19)	121 (81)	.765
**Characteristics related to the GAP**					
GAP territory	0 (0)				
GAP A		790 (60)	122 (15)	688 (85)	<.001
GAP B		533 (40)	122 (23)	411 (77)	
Assessment trajectory	146 (11)				
GAP agent only		245 (19)	129 (53)	116 (47)	<.001
GAP agent → GAP nurse		774 (59)	103 (13)	671 (87)	
IH^b^ nurse → GAP agent		286 (22)	9 (3)	277 (97)	
IH nurse → GAP agent → GAP nurse		18 (1)	3 (17)	15 (83)	

^a^Based on the 1,104 patients who called for an acute health problem and the amount of missing data for this variable.

^b^IH, Info-Health.

Most patients used the GAP service once (84.7%) or twice (11.4%), but others (3.9%) used it up to five times. Most calls were for an acute health problem (83.4%), mainly ear, nose, and throat (ENT) (34.2%), respiratory (13.5%), gastrointestinal (10.5%), and dermatological (10.4%) conditions. GAP agents and nurses suspected an infectious condition in 5.3% of cases. Only 1.0% mentioned two different motives (e.g., paediatric follow-up and medication renewal) during the same call, whereas 5.5% reported symptoms related to two systems (e.g., dermatological and respiratory symptoms).

More patients used GAP A (59.7%) than GAP B (40.3%). Whereas 18.5% spoke to the GAP agent only, 58.5% of users were oriented to the GAP nurse. Almost one-quarter (23.0%) accessed the GAP liaison service after being assessed by an Info-Health nurse.

Agents and nurses (GAP and Info-Health) deemed 81.6% of paediatric patients as needing a medical appointment. However, not all of them received one. In fact, 2.4% were put on a waitlist, 1.7% had to find an appointment on their own, and .6% refused the one offered. Only three (.3%) were directed to an emergency department due to urgent respiratory conditions. Among paediatric users who did not require a medical appointment (18.4%), 36.5% were given other health advice, such as how to self-manage their symptoms (50.1%), and/or were oriented to another health professional (52.8%).

In bivariate analyses (Table 1), we observed significant statistical differences between patients requiring a medical consultation and those who did not regarding age, assignment of a health vulnerability code, main reason for calling, GAP territory, and assessment trajectory. These variables are included in the multivariable model presented below.

### Objective 2: Assess the associations between the characteristics of paediatric patients and the recommendation to have a medical appointment or not

By considering only the variables that were statistically significant in bivariate analyses, we built a multivariable regression model. As displayed in Table 2, younger age groups (especially infants), patients calling for medication renewal or reassessment, users of GAP A, and those who consulted the Info-Health nurse prior to the GAP service were more likely to require a medical consultation. Users calling for general information (e.g., registration to CWL, file update, and form to fill out) and those who spoke exclusively to a GAP agent were less likely to need a medical appointment.

**Table 2.** Multivariable regression model assessing the association between study variables and the need for a medical referral (n = 1,309).

	Adjusted OR^a^	95% CI^b^	*P* value
Age group			
Infancy (0-2)	1.820	1.106-2.997	.019
Early childhood (3-5)	1.538	.855-2.766	.151
Late childhood (6-11)	1.266	.811-1.976	.299
Adolescence (12-17)	Ref.^c^		
Health vulnerability code			
Yes	3.096	.770-12.450	.111
No	Ref.		
Main reason for call			
Paediatric follow-up	.600	.314-1.146	.122
Medication renewal or reassessment	4.306	1.990-18.727	.049
Other	.038	.017-.085	<.001
Acute health problem	Ref.		
GAP territory			
GAP B	.475	.332-.680	<.001
GAP A	Ref.		
Assessment trajectory			
GAP agent only	.303	.194-.474	<.001
IH^d^ nurse → GAP agent	4.938	2.372-10.280	<.001
IH nurse → GAP agent → GAP nurse	.684	.189-2.473	.562
GAP agent → GAP nurse	Ref.		
**Pseudo-R** ^**2**^			.291

^a^OR, odds ratio.

^b^CI, confidence interval.

^c^Ref., reference value.

^d^IH, Info-Health.

## Discussion

Based on our analysis, four factors increased the likelihood of paediatric patients to require a medical consultation after evaluation by the GAP service: (1) being an infant (0-2 years), (2) requesting medication renewal or reassessment, (3) using GAP A, and (4) being assessed by an Info-Health nurse prior to the GAP service. Assumptions can be made to explain these findings. First, infants are more vulnerable to complications (e.g., respiratory distress and dehydration) of undiagnosed or untreated conditions, especially infections (e.g., bronchiolitis, pneumonia, otitis, and gastroenteritis).^18^ Also, because they cannot express their symptoms at that age, it is even more challenging for the parent and the on-line evaluator to have a clear idea of what is happening. Thus, safety concerns may explain why this group is more likely to require a medical appointment than older groups. Furthermore, calls for medication renewal or reassessment might include children who have not had a medical follow-up for a long time and those with clinical deterioration (e.g., behaviour problems) or newly diagnosed conditions (e.g., attention deficit) that require the introduction of a drug, justifying the need for a medical evaluation.

Regarding the GAP territories and entry points, we noticed two important findings. On one hand, paediatric patients relying on GAP A were twice as likely to require a medical appointment than those who used GAP B, even though both are administered by the same health organization (CISSS). In discussions with GAP experts, we learnt that GAP A had more agents and nurses and less staff turnover than GAP B. Also, GAP A had developed strategies earlier in the process to reduce the patients’ waiting time on the phone, including a web form with the possibility of being contacted by an agent the next day. Finally, the number of affiliated medical clinics and appointment slots was higher in GAP A than in GAP B. It is reasonable to say that these factors can not only affect how the GAP works but also the likelihood that patients will use the service again.

On the other hand, while representing 23.0% of GAP users, patients who were redirected from the Info-Health line to the GAP line were five times more likely to require a medical appointment. This can be explained by the fact that Info-Health users almost exclusively call for acute health problems, whereas the GAP deals with various motives, including administrative tasks that do not require a medical appointment (e.g., CWL registration and information about the GAP).^26^ Also, Info-Health nurses only redirect patients who need an appointment to the GAP service, as they are not able to assign one themselves. This reorientation adds a delay for users, who must then repeat some of their information to the GAP agent because computer systems are not linked. To reduce inequities related to GAP trajectories and entry points, improve the fluidity of the process, and optimize the user experience, health leaders need to be aware of these elements and address them.

From the experience in Montérégie, are GAPs a good option for unattached paediatric patients? During a period of high viral circulation, 81.6% of patients were deemed to require a medical evaluation based on a phone call. Without the GAP option, it is conceivable that some of them may have turned to the emergency department had they not found other modalities to access primary care. Also, 18.4% did not need a medical appointment after receiving health advice, including self-management of symptoms, or being reoriented to another health professional. Family physicians might be more available because substantial financial incentives were negotiated to encourage their participation and the provision of appointment slots early in the implementation of GAPs; however, these steps were not taken with other health professionals. Although improving access to doctors is highly valued by families and policy-makers, one might question whether all medical consultations resulting from the GAP service are relevant or convenient. For instance, several minor conditions (e.g., common cold) might be adequately addressed by other health professionals, including pharmacists and NPs. At some point in the GAP deployment, community pharmacists were involved and agreed to see unattached patients for minor conditions and renewal of common medications; our study demonstrated their contribution, as 15.2% of users were oriented to a pharmacist. However, none of the paediatric GAP users under study were referred to a NP. The limited number of general NPs (n = 29) and the absence of paediatric NPs in our study’s jurisdiction may explain this situation to some extent. Finally, because there is no formalized pathway connecting the GAP service to other health professionals (e.g., physiotherapists), patients must see a doctor to get a referral. In this context, to improve the relevance of care and reduce health costs related to unnecessary medical consultations, we encourage decision-makers to discuss incentives and develop trajectories with other health professionals working in PHC to be integrated into the functioning of the GAP service.

Increasing access to acute care for children via the GAP service is admirable, but it does not address this population’s need for preventive services, nor does it offer the continuity of care that healthcare organizations (e.g., American Academy of Pediatrics and Canadian Paediatric Society) advocate.^13,14^ Security is also a concern, as the initial assessment and decisions are made in part by an administrative clerk with no medical background using an algorithm, although it is assumed that agents contact a nurse for advice when in doubt. Phone assessments are also limited by the fact that they rely solely on the parent’s (or patient’s) observations and on the evaluator’s attention and understanding of the problem. Moreover, we were not able to validate how many parents were advised to see a doctor promptly should their child’s condition deteriorate, whether the GAP service assigned them an appointment or not. Lastly, since no uniform training regarding paediatric conditions is offered to GAP employees, decision-making tools must be optimized and such training must be standardized.

Our study presents some limitations. First, it was based on available clinical-administrative data that were not collected for research purposes. Consequently, data were missing for some variables (e.g., system affected in the event of an acute problem), thereby limiting our analyses. Second, to simplify data analysis, we considered only one call per patient. Although this may have affected clinical variables (e.g., main reason for calling and system affected in the event of an acute health problem) and characteristics related to the GAP, sociodemographic factors were not affected. Given that only 15% of patients called more than once, we consider this bias to be minimal. Third, our database did not allow us to know what happened after the call, whether the patient showed up at their appointment or went to the emergency department. Furthermore, our results may have differed had the study been conducted at another time of year when viruses are less active in the population. Finally, because we only documented GAPs in one region, our findings may not be generalizable to other GAPs in the province of Quebec.

## Conclusion

Though imperfect, the implementation of single points of access such as Quebec’s GAP service is a first step to improve access to primary care for unattached patients. Challenges concerning the relevance of medical referrals, equity regardless of entry point, and safety throughout the assessment process need to be addressed. To guide decision- and policy-makers, we recommend (1) offering standardized staff training regarding paediatric conditions, (2) optimizing decision-making tools, (3) integrating trajectories involving other health professionals, and (4) clarifying with the population the role and the right way to use the GAP service. Finally, we recommend that qualitative studies be conducted to better understand the challenges and facilitators regarding the GAP process from the perspectives of patients, field workers, and managers.
